# Deletion of the *MBP1* Gene, Involved in the Cell Cycle, Affects Respiration and Pseudohyphal Differentiation in Saccharomyces cerevisiae

**DOI:** 10.1128/spectrum.00088-21

**Published:** 2021-08-04

**Authors:** Xiaoling Chen, Zhilong Lu, Ying Chen, Renzhi Wu, Zhenzhen Luo, Qi Lu, Ni Guan, Dong Chen

**Affiliations:** a National Engineering Research Center for Non-Food Biorefinery, Guangxi Academy of Sciences, Nanning, Guangxi, People’s Republic of China; Broad Institute

**Keywords:** respiration, *MBP1* gene, *Saccharomyces cerevisiae*, ethanol fermentation, pseudohyphal differentiation

## Abstract

Mbp1p is a component of MBF (MluI cell cycle box binding factor, Mbp1p-Swi6p) and is well known to regulate the G_1_–S transition of the cell cycle. However, few studies have provided clues regarding its role in fermentation. This work aimed to recognize the function of the *MBP1* gene in ethanol fermentation in a wild-type industrial Saccharomyces cerevisiae strain. *MBP1* deletion caused an obvious decrease in the final ethanol concentration under oxygen-limited (without agitation), but not under aerobic, conditions (130 rpm). Furthermore, the *mbp1*Δ strain showed 84% and 35% decreases in respiration intensity under aerobic and oxygen-limited conditions, respectively. These findings indicate that *MBP1* plays an important role in responding to variations in oxygen content and is involved in the regulation of respiration and fermentation. Unexpectedly, *mbp1*Δ also showed pseudohyphal growth, in which cells elongated and remained connected in a multicellular arrangement on yeast extract-peptone-dextrose (YPD) plates. In addition, *mbp1*Δ showed an increase in cell volume, associated with a decrease in the fraction of budded cells. These results provide more detailed information about the function of *MBP1* and suggest some clues to efficiently improve ethanol production by industrially engineered yeast strains.

**IMPORTANCE** Saccharomyces cerevisiae is an especially favorable organism used for ethanol production. However, inhibitors and high osmolarity conferred by fermentation broth, and high concentrations of ethanol as fermentation runs to completion, affect cell growth and ethanol production. Therefore, yeast strains with high performance, such as rapid growth, high tolerance, and high ethanol productivity, are highly desirable. Great efforts have been made to improve their performance by evolutionary engineering, and industrial strains may be a better start than laboratory ones for industrial-scale ethanol production. The significance of our research is uncovering the function of *MBP1* in ethanol fermentation in a wild-type industrial S. cerevisiae strain, which may provide clues to engineer better-performance yeast in producing ethanol. Furthermore, the results that lacking *MBP1* caused pseudohyphal growth on YPD plates could shed light on the development of xylose-fermenting S. cerevisiae, as using xylose as the sole carbon source also caused pseudohyphal growth.

China produces approximately 3.8 million tons of sugarcane molasses annually as a byproduct of sugar processing. This molasses contains approximately 45 to 50% (wt/wt) fermentable sugars that can be converted to ethanol by fermentation. Saccharomyces cerevisiae is widely used for ethanol production from molasses (1, 2) because of its robustness under industrial fermentation conditions (3). However, numerous inhibitors in molasses and the high osmolarity conferred by sugars still greatly affect yeast cell growth and ethanol production (4), and high concentrations of ethanol during the late stage of fermentation are also harmful to cells (5). Therefore, yeast strains with high performance, such as high tolerance, rapid growth, which can shorten the fermentation time, and high ethanol productivity, are highly desirable for industrial-scale ethanol production.

Nitrogen and other nutrient levels early in fermentation are factors that are extremely important in determining yeast cell growth and a successful fermentation outcome (6–8). The nutrients of which a cell can be starved include carbon and nitrogen (9). With sufficient nutrients, wild-type S. cerevisiae cannot form pseudohyphae, but when cells are starved for nitrogen (10, 11), carbon (12), and especially fermentable sugars (13), they switch from a yeast form to a filamentous pseudohyphal form (11) and invade into agar (13). Filamentous/invasive growth allows sessile yeast cells to forage for scarce nutritional resources (11, 12).

We previously isolated two industrial strains, MF01 and MC15, from 1-year-old sugar mill waste in Nanning, China. MF01 was found to possess better fermentative capabilities and has been used for industrial-scale ethanol production with an annual output of 50,000 tons but showed slower growth than MC15 (14). Comparative genomics between these strains showed that several candidate regulatory genes are potentially associated with ethanol fermentation (14). Among these genes, this study focused on *MBP1*, a gene involved in the cell cycle.

Although *MBP1* is well known for its role in the cell cycle, little is known about its function in ethanol fermentation, especially in industrial strains. In this study, we deleted *MBP1* in the MF01 strain and focused on the phenotypes and ethanol productivity of the resulting *mbp1*Δ strain. We found that *MBP1* deletion decreased the fermentation ability and unexpectedly caused pseudohyphal differentiation and respiration defects. We report here pseudohyphal differentiation of S. cerevisiae cultured in rich medium (yeast extract-peptone-dextrose; YPD) to our knowledge. We further present several hypotheses that might help to understand the mechanisms underlying our results.

## RESULTS

### Deletion of *MBP1* decreases fermentability in molasses medium.

**FIG 1:**
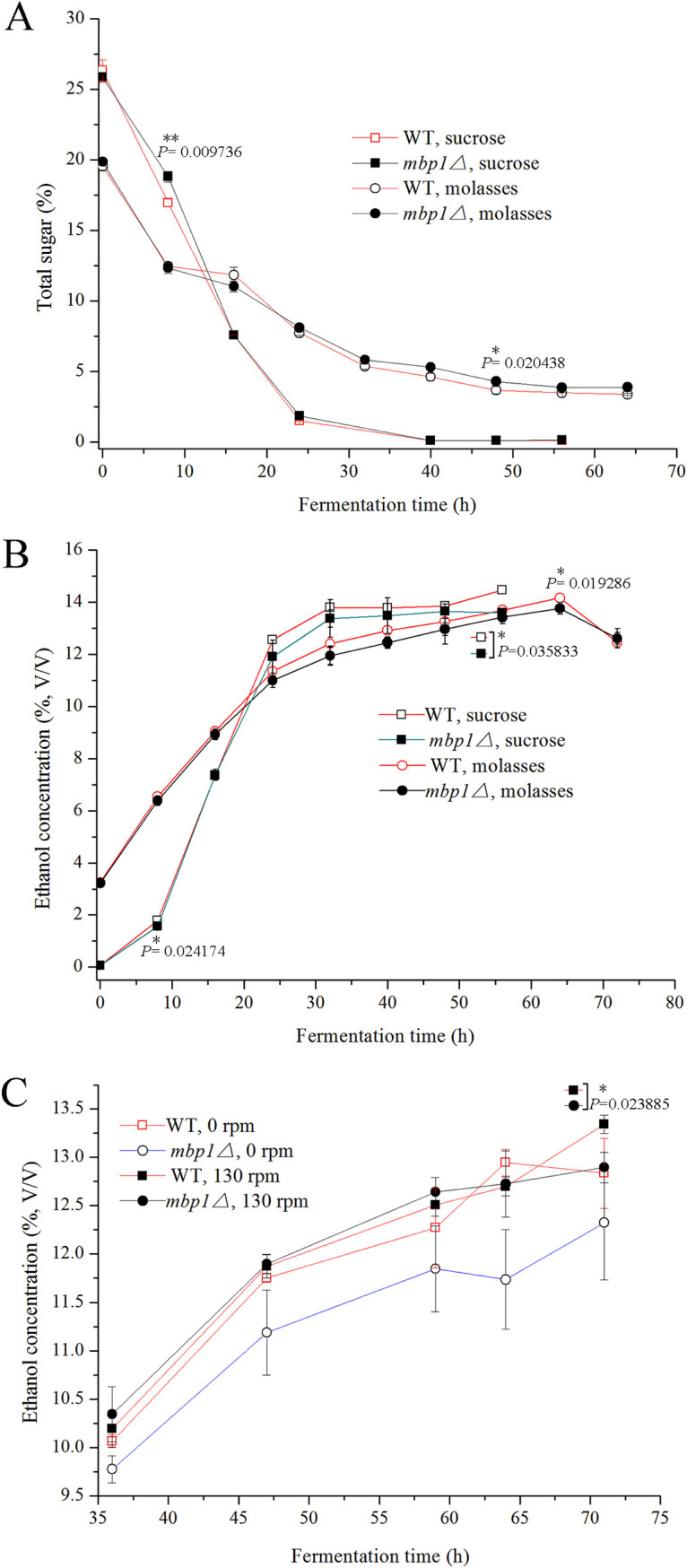
Fermentability analysis. For the determination of total residual sugar (A) and ethanol production with different carbon sources (B), samples were collected as described in Materials and Methods. (A) One part of 6 M HCl was added to 10 parts of the fermentation broth and boiled for 15 min. After cooling quickly to room temperature, 1.2 parts of 20% NaOH was added. Total sugar was estimated by dinitrosalicylic acid method (62). Data shown represent mean ± standard deviation (SD) of data from three replicate fermentations. (B) The concentration of ethanol was analyzed using an Agilent gas chromatograph, model 6890N (Agilent Technologies Inc., USA) with a flame ionization detector. Data shown represent mean ± SD of data from three replicate fermentations. (C) For the determination of ethanol production under different aeration conditions, cells were grown and fed with molasses medium as described in Materials and Methods. Equivalents of the cells were then cultured with shaking (130 rpm) and without shaking, respectively. Samples were taken at 36, 47, 59, 64, and 72 h after feeding. Data shown represent mean ± SD of three replicate fermentations.

We obtained a diploid mutant lacking *MBP1* by the method described in Materials and Methods. To examine whether the lack of *MBP1* alters the fermentation ability of S. cerevisiae, we tested the ability of yeast to ferment sucrose and sugarcane molasses. Upon fermentation with sucrose as the sole carbon source, the total residual sugar in the fermentation broth of the mutant (*mbp1*Δ) and the wild-type (WT) strains decreased from 25.86% ± 0.33% and 26.35% ± 0.76% to 0.11% ± 0.00% and 0.11% ± 0.00%, respectively, and the utilization rate of sucrose by both strains was 99.6% (Fig. 1A). The maximum ethanol concentration obtained with *mbp1*Δ using sucrose was 13.65% ± 0.03%, which was slightly lower than that of the WT, 14.47% ± 0.05% (Fig. 1B). However, when molasses was used as the sole carbon source, the total sugar with *mbp1*Δ and WT decreased from 19.87% ± 0.14% and 19.55 ± 0.26% at the beginning of feeding (0 h) to 3.88% ± 0.15% and 3.37% ± 0.07% at 64 h, respectively, and the utilization rate of molasses by *mbp1*Δ was 80.5%, lower than that by the WT (82.8%) (Fig. 1A). As shown in Fig. 1B, the maximum ethanol concentration with *mbp1*Δ was 13.77% ± 0.23%, which was slightly lower than that of WT (14.16% ± 0%).

### *MBP1* deletion causes respiration defects and obviously decreases ethanol production under oxygen-limited conditions.

Since a lack of *MBP1* might cause decreased adaptability in molasses, we paid more attention to fermentation in molasses medium under different aeration conditions. When molasses medium was cultured with shaking (130 rpm), there was little difference in the ethanol concentration between the mutant and parent strains. In contrast, when the medium was cultured without shaking, the ethanol production by the mutant was significantly lower than that by the parent strain, with 2.9%, 4.8%, 3.5%, 9.3%, and 4.0% decreased, respectively, at each measurement time (36, 47, 59, 64, and 71 h). The ethanol concentration of *mbp1*Δ was significantly higher under aerobic conditions (130 rpm) than under oxygen-limited conditions (0 rpm). However, ethanol production by the parent strain showed a limited difference between the two conditions, although there was slight enhancement under aerobic conditions (except at 64 h) (Fig. 1C).

Considering this surprising difference under different aeration conditions, we checked the respiratory intensity of the strain. The *mbp1*Δ strain showed obvious defects in respiratory intensity displayed by the reduction of 2,3,5-triphenyltetrazolium chloride (TTC), which was exceptionally apparent under aerobic conditions; this was 16.79% and 64.52% compared to that of the parent strain under aerobic conditions and with insufficient oxygen supply, respectively. Interestingly, under oxygen-limited conditions, the mutant showed 1.8 times the respiratory intensity under aerobic conditions, whereas this was 47.3% for the WT under aerobic conditions (Table 1).

**TABLE 1:** Respiration intensity^*a*^

Strain	Rotation speed	
	130 rpm^*b*^	0 rpm^*c*^^,^^*d*^
WT^*e*^	1.31 ± 0.49	0.62 ± 0.04
*mbp1*Δ^*f*^	0.22 ± 0.02	0.40 ± 0.02

a Respiration intensity was presented as OD_490_. The data represent the TTC absorbance after 4 h incubation.

b Statistical comparison between WT and *mbp1*Δ: ns, *P *= 0.188508.

c Statistical comparison between WT and *mbp1*Δ: *, *P *= 0.026926.

d Although yeast cells produce a lot of carbon dioxide, cultivation without shaking does not guarantee that the medium was totally anaerobic (65). Thus, we indicated this cultivation as oxygen-limited condition.

e Statistical comparison for WT between 130 rpm and 0 rpm: ns, *P *= 0.314887.

f Statistical comparison for *mbp1*Δ between 130 rpm and 0 rpm: ***, *P *= 0.000243.

### Deletion of *MBP1* alters colony morphology and causes invasive growth.

**FIG 2:**
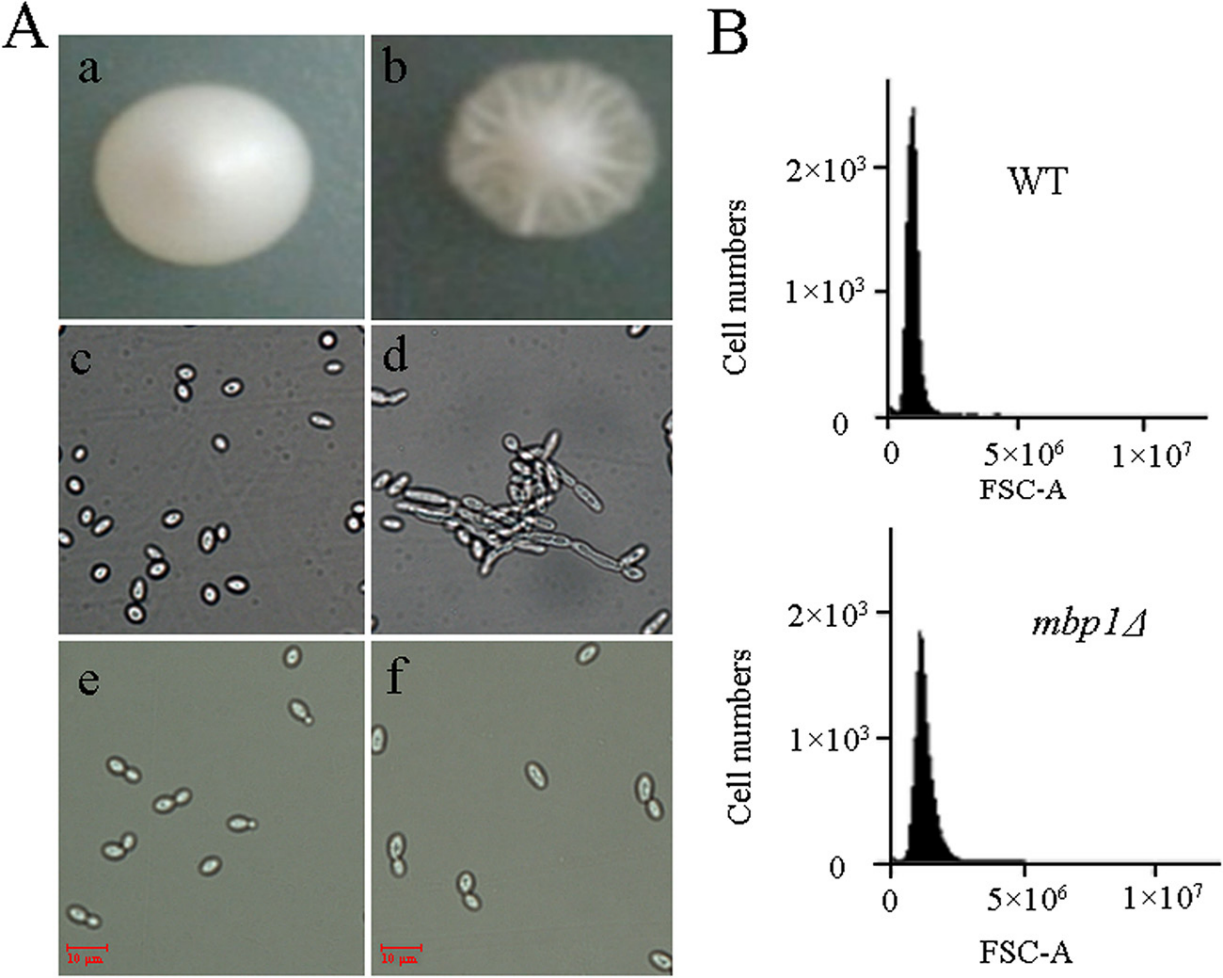
Morphology and size analysis. (A) Wild-type (a and c) and *mbp1*Δ (b and d) cells were incubated for 3 to 5 days on YPD plates and visualized on petri plates (a and b) and then picked from the edge of colony and viewed by microscopy (c and d). Wild-type (e) and *mbp1*Δ (f) cells cultured in liquid medium were also checked by microscopy. (B) Synchronized cells were grown to exponential growth, diluted to 1 × 10^6^ cells/ml, and analyzed (10,000 events collected per sample) by FSC using an Accuri C6 flow cytometer (Becton, Dickinson, USA) at the medium flow rate. Cell size was estimated by forward scatter area (FSC-A). Each FSC was determined from an average of data from duplicate experiments.

Unexpectedly, the deletion of *MBP1* resulted in surface invaginations and roughness at the periphery of the colony on the YPD plate, in contrast to normal smooth, lustrous colony surfaces (Fig. 2A, panels a and b). Microscopic examination revealed that *mbp1*Δ cells became long and grew as chains of cells in a filament, whereas the WT cells were separated into ellipsoidal yeast cells (Fig. 2A, panels c and d). Therefore, we further examined their growth in liquid medium. Microscopic analysis showed that *mbp1*Δ cells were elongated (Fig. 2A, panel f). The forward angle scattering (FSC) of WT and *mbp1*Δ was 988,262 ± 100,525 and 1,177,935 ± 83,402, respectively (Fig. 2B), showing an increase in mutant cell volume.

### Deletion or overexpression of *MBP1* caused a slow growth.

**FIG 3:**
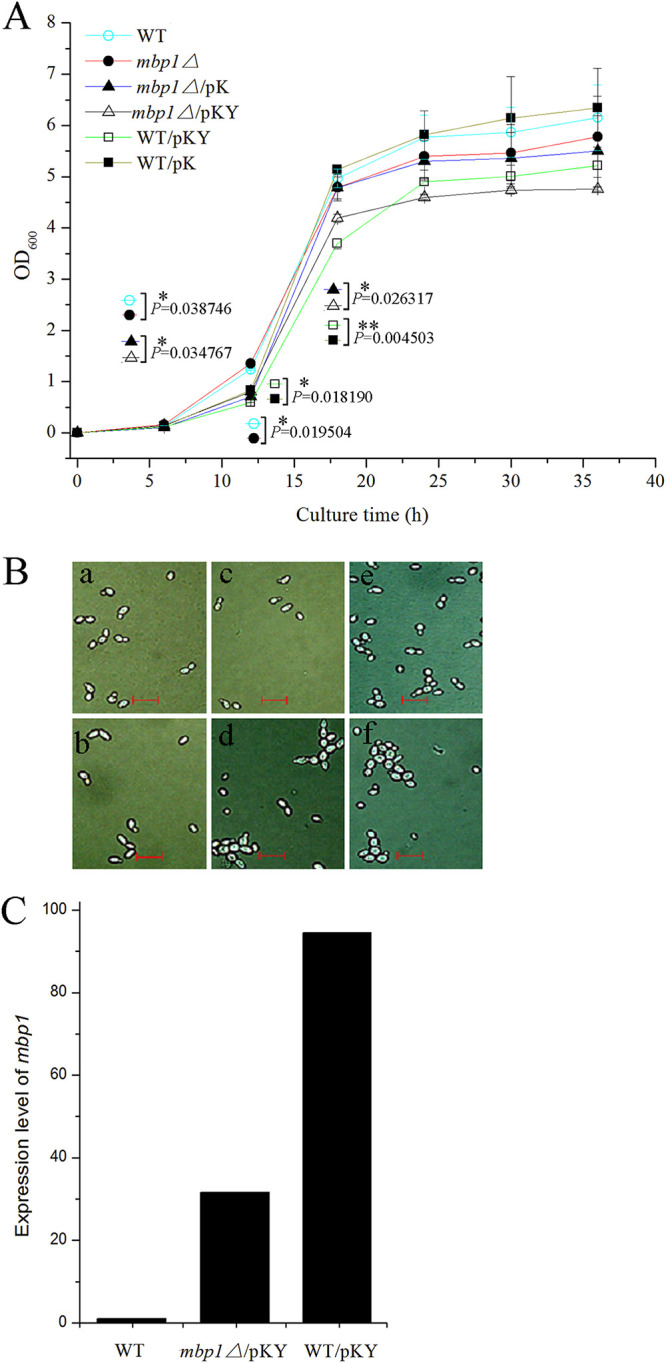
Deletion or overexpression of *MBP1* causes slow growth and altered cell morphology. (A) After cells were synchronized, the number of cells in each sample was determined by microscopy and equivalent of cells were harvested by centrifugation, resuspended in sterile water, and shifted to YPD containing 2% galactose, at a final concentration of 1 × 10^6^ cells/ml. Samples were taken at 6-h intervals and absorbance was measured at 600 nm. WT/pK and *mbp1Δ*/pK harbored pYes/kanMX, and WT/pKY and *mbp1Δ*/pKY harbored pYes/kanMX/*mbp1*. In these vectors, *MBP1* expression was controlled by *GAL1* promoter. Data shown represent mean ± SD of data from three independent biological replicates. (B) Synchronized cells were incubated for 30 h in YPD plus 2% galactose and determined by microscopy. a: WT, b: *mbp1*Δ, c: *mbp1*Δ/pK, d: *mbp1*Δ/pKY, e: WT/pK, f: WT/pKY. Scale bars, 20 μm. (C) Synchronized cells were harvested after 18 h of cultivation in YPD plus galactose. Gene transcript levels were determined by the 2^−ΔΔCt^ method (63) using *PDA1* (Table 2) as a reference gene for normalizing *MBP1* expression levels and WT as a reference sample.

Two different vectors (pK and pKY) were constructed and introduced into the WT and *mbp1*Δ strains. Cells overexpressing *MBP1* (transformed pKY; Fig. 3B, panels d and f) were larger than the controls (transformed with pK; Fig. 3B, panels c and e), and this manifested as aggregation. Furthermore, at 36 h, the optical density at 600 nm (OD_600_) value of the *mbp1*Δ strain was 94.0% that of WT, whereas those of WT/pKY and *mbp1*Δ/pKY were 82.2% and 86.5% those of WT/pK and *mbp1*Δ/pK, respectively (Fig. 3A). In the platform growth period, the absorbance (OD_600_) of WT/pKY was lower than that of WT/pK or WT, and the growth curves of both strains were close to parallel; *mbp1*Δ/pKY and *mbp1*Δ/pK followed the same patterns. Since overexpression and deletion cells were bigger than their respective controls, their cell density was even lower than that of the controls, based on comparisons with the OD_600_ data. The similar morphology (Fig. 3B, panels d and f) and growth rate (Fig. 3A) between WT/pKY and *mbp1*Δ/pKY cells might result from the overexpression of *MBP1*. To confirm this hypothesis, quantitative reverse transcription (qRT)-PCR analysis was performed. The relative expression levels of *MBP1* were 32 and 94 in *mbp1*Δ/pKY and WT/pKY, respectively (Fig. 3C), corresponding to their OD_600_ value at 18 h (Fig. 3A), which was consistent with our hypotheses. Taken together, we concluded that both the overexpression and deletion of *MBP1* caused slow growth (Fig. 3A) and an increase in cell volume (Fig. 2B; Fig. 3B).

### *MBP1* deletion causes growth inhibition in the presence of stress.

**FIG 4:**
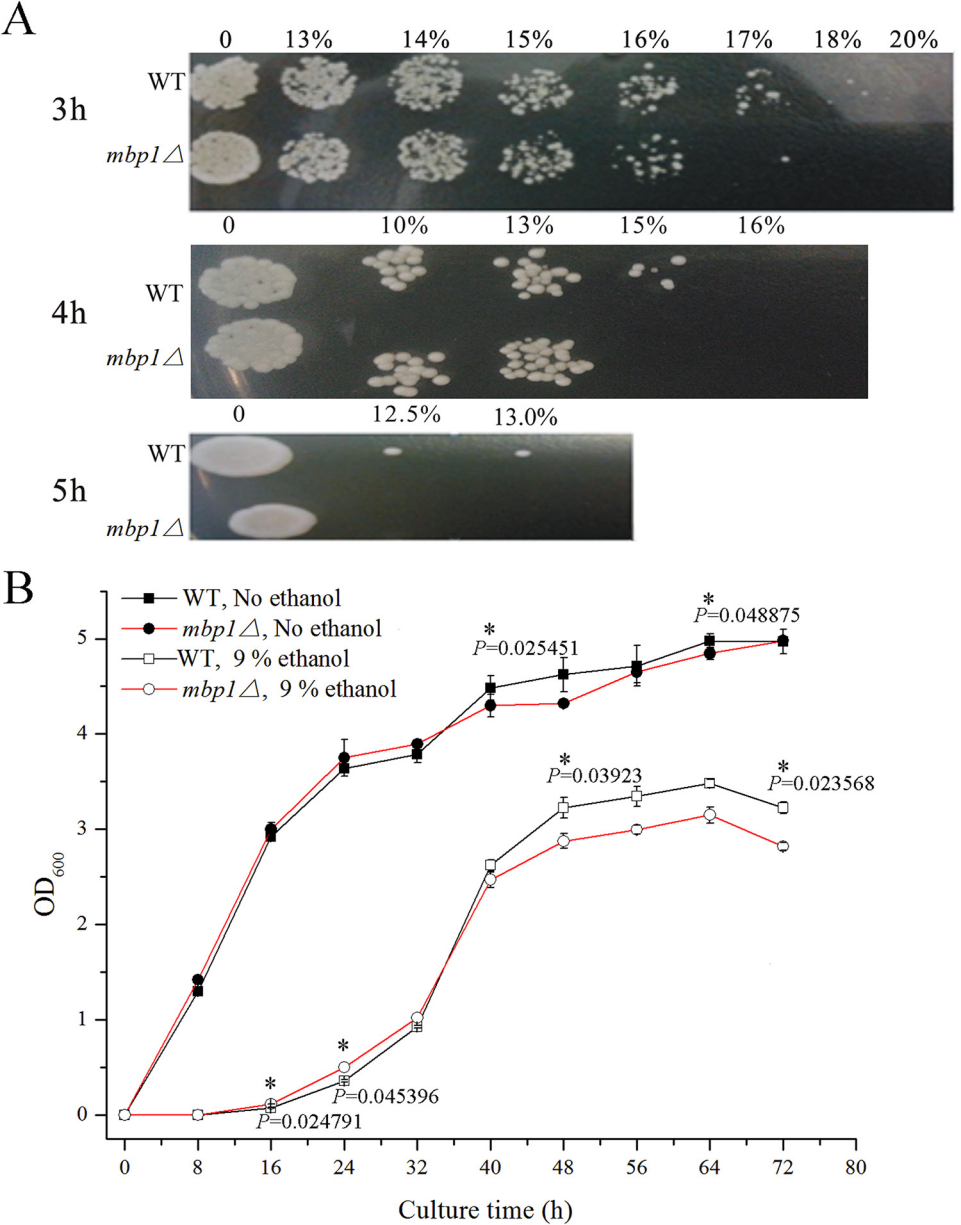
The mutant showed a tolerance weaker than that of the WT. (A) A total of 2 × 10^6^ overnight cells were inoculated into 1 ml YPD with ethanol at different concentrations and cultured for 3, 4, and 5 h, respectively. A total of 2 μl of each treated culture was spotted in rows on YPD plates. After the spots dried, the plates were incubated for 2 to 3 days. The treatment time and strain are indicated at the left of each panel, while the concentrations of ethanol are listed above. (B) A total of 1 × 10^6^ overnight cells were reinoculated into 10 ml YPD and YPD plus 9% ethanol, respectively, for growth. Samples were taken at 8-h intervals. Data shown represent mean ± SD of data from two replicate fermentations.

**FIG 5:**
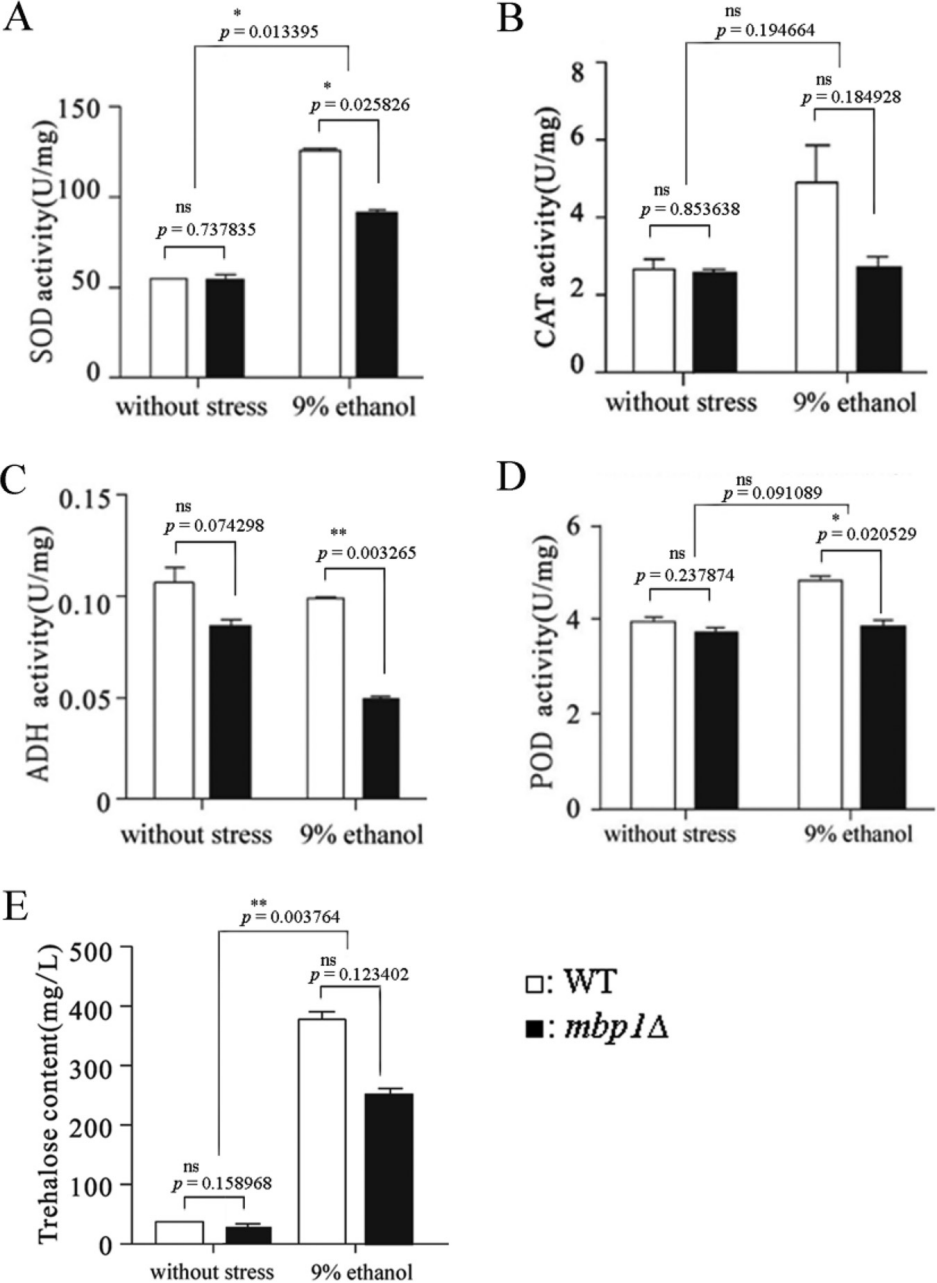
Content of trehalose and activity of enzymes involved in tolerance. (A to D) Overnight cells were cultured in 50 ml YPD with or without 9% ethanol in the final concentration of 1 × 10^6^ cells/ml and harvested by centrifugation after 48 h cultivation. Data shown represent mean ± SD of data from two or three replications. (E) After 54 h cultivation with or without ethanol, equal weight (38.5 g) of cells in each sample was collected. Trehalose was extracted using 0.5 M trichloroacetic acid and measured by the anthrone method (64). The amount of trehalose was determined using a standard curve of amounts of trehalose plotted against OD_620_. Data shown represent mean ± SD of data from two replications.

**FIG 6:**
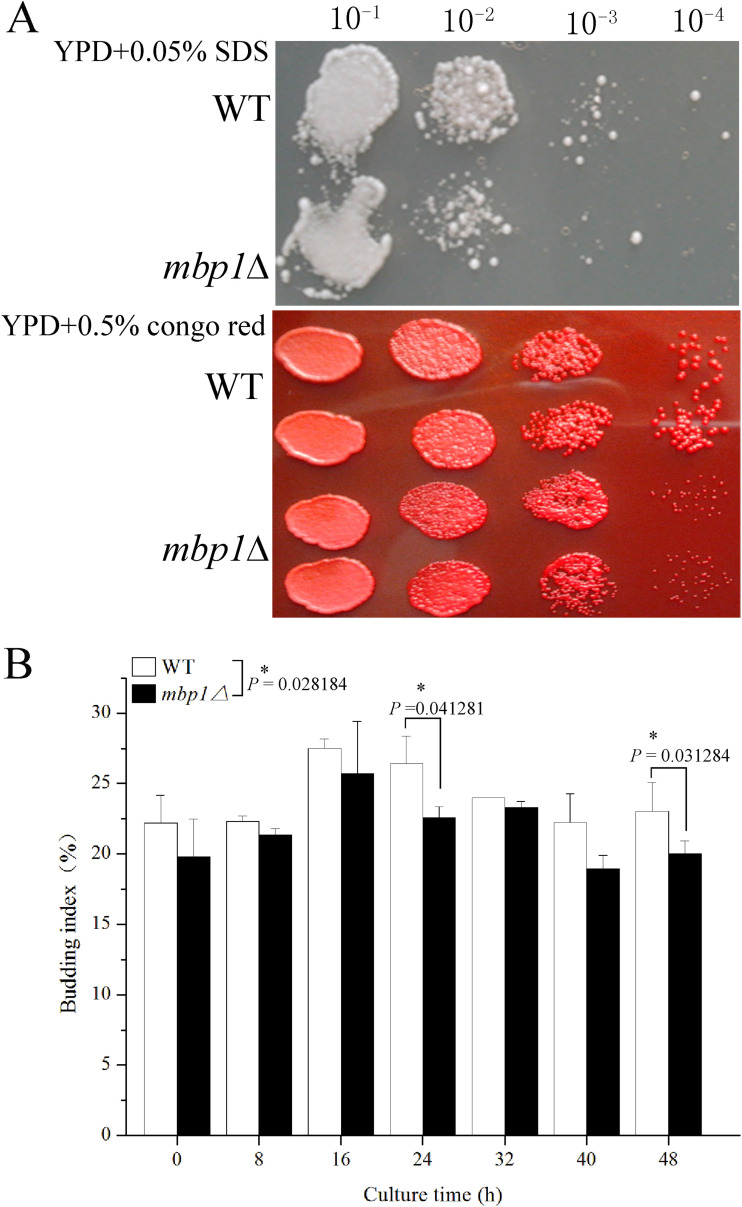
Sensitivity to cell wall inhibitor (A) and budding index (B). (A) Cells were grown in YPD medium to log phase (2 × 10^7^ cells/ml) and serially diluted 10-fold, and 2 μl each of the 10-fold dilutions of cultures for each strain was spotted onto solid medium supplemented with inhibitor and cultured for 2 to 3 days. The strains and dilution times are indicated at the left and the top of the picture, respectively. (B) Synchronized cells were inoculated into 5 ml YPD with the final concentration 2 × 10^6^ cells/ml. Samples were taken at 8-h intervals and the number of budded cells (*N*_budded_) and total cells (*N*_total_) in each sample was determined. Budding index is equal to *N*_budded_/*N*_total_. Data shown represent mean ± SD of data from two replicate fermentations.

To provide more proof that *MBP1* contributes to cellular fitness, we determined the strains’ tolerance to ethanol. First, the strains were treated with ethanol at different concentrations for different times. It was found that *mbp1*Δ showed tolerance weaker than that of the WT after 3 h of treatment with 17% ethanol, as well as with longer treatment with less ethanol (Fig. 4A). Their growth rates were then checked. Both strains showed similar growth without ethanol in the liquid medium. In contrast, the OD_600_ of *mbp1*Δ was obviously lower than that of the WT after 40 h of culture with 9% ethanol (Fig. 4B). Taken together, we propose that a lack of *MBP1* greatly affects the fermentation rate, especially in the late stage of fermentation, since biomass content governs the fermentation rate (6). To gain more insight into cellular fitness, we determined the content of trehalose and the activity of enzymes involved in tolerance. Consistent with tolerance, after culture with 9% ethanol, the contents of trehalose and the activities of CAT, POD, SOD, and ADH in *mbp1*Δ were 49%, 80%, 24%, 37%, and 73% lower, respectively, than those in the WT, but no significant difference was observed between the strains without ethanol (Fig. 5). Furthermore, we assessed the sensitivity of yeast cells to cell wall inhibitors and found that *mbp1*Δ was more susceptible to SDS and Congo red (Fig. 6A). These results indicate that *MBP1* plays a role in maintaining cell wall integrity, which helps to optimize the cell survival response to stress and the fermentation ability.

## DISCUSSION

### Clues for industrial strain development.

S. cerevisiae is an industrial strain that is important for bioethanol production. Numerous studies have been conducted to improve the performance of ethanol production (15–18). Most studies have focused on laboratory strains (19, 20), which might contain many naturally occurring mutations during cultivation (21), thus reducing reference value for industrial strains. The merits of industrial yeast have also been reported (22).

This study focused on wild-type industrial strains and uncovered the role of *MBP1* in ethanol fermentation. Deletion of *MBP1* decreases the utilization rate of molasses. The difference in the utilization rate of molasses might result from the difference in adaptation to a complex environment between the mutant and its parent strain, since molasses medium was more complex than sucrose medium. Furthermore, *MBP1* loss decreased the content of trehalose and the activity of enzymes involved in tolerance. Trehalose accumulation is responsible for sustaining cell viability and is highly correlated with ethanol production (6). Our results suggest that *MBP1* participates in the regulation of genes involved in trehalose biosynthesis and enzymes related to tolerance. Unexpectedly, *mbp1*Δ showed filamentous growth on YPD plates, generally caused by the starvation of nitrogen (10, 11) or carbon (12, 13). In addition, filamentous growth was also observed in a recombinant industrial strain using xylose as the sole carbon source (unpublished data). These results could shed light on the development of ethanol- and xylose-fermenting S. cerevisiae with better performance, which is important for developing an economical microbial conversion process of lignocellulosic biomass into bioethanol (23).

Several kinds of *MBP1*-based approach could potentially be used for the construction of yeast strain with high performance, such as CRISPR-Cas9 and homologous recombination, by which two strains with high performance were obtained via replacement of *PHO4* (14) and *CDC15* (unpublished data), respectively. Further research could check whether changes in the expression levels of Mbp1p could improve the ethanol productivity in a complex environment, although the overexpression caused a slow growth in medium without stress/inhibitor (Fig. 3A).

### Phenotype affected by *MBP1* deletion.

To the best of our knowledge, the phenotypic consequence of *MBP1* deletion was first described by Bean et al. (24) in a W303 strain background. The *mbp1*Δ mutant showed a 20% increase in cell volume, associated with a 5% increase in budded cells among haploid yeast during exponential growth (24). However, according to Porter et al. (25), the loss of *MBP1* in haploid yeast leads to a decrease in the proportion of budded cells in the first doubling time but an increase during 1.3 to 1.8 doubling times. Herein, the *mbp1*Δ mutant showed a 19.2% increase in cell volume (Fig. 2A, panels e and f; Fig. 2B), as reported by Bean et al. (24), but a decrease in budding index throughout the test (Fig. 6B), consistent with the report that large cell size impairs cell proliferation (26). *MBP1* loss may delay cell division relative to cell growth and cause cells to be arrested in G_1_ and grow to their maximal size, since cell size is a sensitive indicator of cell cycle progression overall (27). More phenotypic differences have been reported. For example, some studies found that *mbp1*Δ *swi4*Δ strains failed to survive (24, 28), whereas another study obtained a *swi4*Δ *mbp1*Δ strain (29). Moreover, no apparent growth defects were observed in both *swi4*Δ *mbp1*Δ and *mbp1*Δ haploid strains in a BY4741 strain background (29), but minor growth defects were observed in *mbp1*Δ*/mbp1*Δ Candida albicans cells (30). In this study, the lack of *MBP1* altered colony morphology (Fig. 2A, panel b) and caused invasive growth (Fig. 2A, panel d). The reasons for these discrepancies related to *MBP1* deletion were likely due to different genetic backgrounds. For example, some laboratory strains (S288C and W303) fail to form pseudohyphae, resulting from a naturally occurring mutation in the *FL08* gene (21).

Significantly, *MBP1* deletion caused a slow growth, and so did the overexpression of *MBP1*. The slow growth phenotype could not be rescued by the overexpression, which might be attributed to an overdose of *MBP1*. In addition, previous work has found that high overexpression of *MBP1* is lethal (31), unlike our results. The difference in viability of cells after overexpression of *MBP1* may also result from the difference in *MBP1* dosage or genetic background.

### The role of *MBP1* in pseudohyphal growth.

To the best of our knowledge, the requirements for pseudohyphal growth were first described in detail based on nitrogen starvation, an **a**/α diploid form, and BUD genes that regulate the budding pattern (11). However, contradictory results have been observed. For example, both haploid and diploid S. cerevisiae can form pseudohyphae in response to carbon limitations in the presence of sufficient nitrogen (12). Similarly, fermentable sugar depletion also causes invasive growth in haploid yeasts (13). In this study, the homozygous diploid mutant (*mbp1*Δ) unexpectedly showed invasive pseudohyphal growth on YPD plates (Fig. 2A, panels c and d). The fundamental cellular basis for invasive pseudohyphal growth could be assumed.

The processes involved in the filamentous phase consist of polar budding, cell elongation, incomplete cell separation, and invasive growth (11) and are regulated by multiple pathways (32). Theoretical considerations predicted that *MBP1* was possibly associated with a gene that links it to filamentation-regulated genes. Some results support our hypothesis. First, deletion of genes, involved in the cell cycle and regulated by MBF, cause abnormal budding (33). Second, MAPK induces cell elongation by regulating *CLN1* (34), a gene essential for pseudohyphal growth (35, 36). In addition, there is some interaction between *MBP1* and *CLN1* (37). Taken together, it might be possible that *MBP1* serves as a transcriptional repressor, repressing the transcription of *CLN1*.

However, since Mbp1p/MBF regulates many genes involved in the cell cycle (24, 28, 30, 32, 33, 38–40), *MBP1* may associate with another gene linked to the filamentous phase, for example, *CLN3*. *CLN3* has an antagonistic effect on pseudohyphal growth (35), and the inhibitory effect might be mediated through *MBP1*. Support for this hypothesis includes the interaction between them; *CLN3* is an activator of MBF (39), and the role of *CLN3* in the regulation of cell size and budding is partly dependent on MBF (39). Further evidence for their interaction might come from our results indicating that *mbp1*Δ showed an increase in cell volume (Fig. 2A, panels e and f; Fig. 2B) and a decrease in the budding index (Fig. 6B).

### Cross talk between filamentous growth and cell wall integrity pathway.

Filamentous growth is a behavior more complex and globally regulated than is currently appreciated (32) and is regulated by MAPK pathways both positively and negatively (34). Cross talk among filamentous growth (FG) and other MAPK pathways, such as the pheromone response (32, 41, 42) and the high-osmolarity glycerol (32, 42), have been reported. Our results showed cross talk between FG and the cell wall integrity pathway (CWI) based on invasive growth (Fig. 2A) and sensitivity to cell wall inhibitors (Fig. 6A) in the *mbp1*Δ mutant. Further evidence is provided by the strong genetic interactions between *MBP1* and *SSE1* involved in CWI (29).

### The effect of *MBP1* deletion on respiration.

Although there was little difference in the final ethanol concentration between *mbp1*Δ and WT under aerobic conditions, a marked difference was observed under the condition of insufficient oxygen supply. Interestingly, the respiratory intensity of the mutant was stronger under oxygen-limited conditions than under aerobic conditions, whereas that of the parent strain was obviously lower. Nevertheless, the respiratory intensity of the mutant strain was lower than that of the parent strain under both conditions. Although the mechanism behind this phenomenon remains unclear, it could be speculated. The ability to grow under aerobic and anoxic conditions is one of the most relevant metabolic features of S. cerevisiae, and cells can obtain energy in both conditions by switching metabolism between respiration and fermentation. The switch between fermentation and mitochondrial respiration is regulated by glucose availability (43). The functionality of mitochondria is dependent on the carbon source (44), and mitochondrial respiration is regulated by *SNF1* depending on the concentration of glucose (45, 46). In addition, *SNF1* regulates MBF-dependent transcription (47, 48) and cyclin *CLB5* (48, 49), which is also regulated by MBF (33). Taken together, we surmise that *MBP1* might affect respiration by interacting with *SNF1*. Further evidence for their interaction is demonstrated by the previous report that *SNF1* is involved in invasive growth (13) and affects cell integrity (50), as well as our similar results caused by the *MBP1* deletion (Fig. 2A; Fig. 6A). However, *MBP1* might affect respiration with other genes linked to mitochondria, since it has been reported that *MBP1* regulates the activity of genes involved in mitochondria, such as YDR263C (*DIN7*) (51, 52) and YMR307W (*GAS1*) (51).

### Conclusion.

*MBP1* deletion caused a decrease of tolerance and an obvious decrease in the final ethanol concentration under oxygen-limited conditions. This provided clues regarding the role of *MBP1* in fermentation and could help to efficiently improve ethanol production by industrially engineered yeast strains. Furthermore, we are not aware of any prior reports of pseudohyphal differentiation of S. cerevisiae cultured in rich medium. Although the cellular mechanisms underlying the pseudohyphal growth were not clear, we proposed several hypotheses that were helpful for uncovering the mechanisms. Nevertheless, our hypotheses about the interaction between *MBP1* and other genes needed to be tested. Since filamentous growth was also observed in the yeast using xylose as the sole carbon source (unpublished data), our results could also shed light on the development of xylose-fermenting S. cerevisiae.

## MATERIALS AND METHODS

### Strain and culture conditions.

Strain MF01 (*MAT***a**/*MAT*α) is a wild-type diploid S. cerevisiae. Unless otherwise stated, the yeast strain was grown in YPD (2% Bacto peptone, 1% yeast extract, and 2% dextrose) at 30°C, with shaking (180 rpm), or YPD solidified with 2% agar.

### Preparation of haploid strains.

For preparation of haploid strains, 0.1 ml of overnight cells was spread on a plate for sporulation (0.82% sodium acetate, 0.1% glucose, 0.25% yeast extract, 0.18% KCl, 2% agar) for 4 to 5 days. The spores were scraped and rinsed from the plate into sterilized water, collected by centrifugation, and washed twice with 0.85% NaCl. The pellets were resuspended in lysis buffer (7 mM Tris-HCl [pH 8.0], 1% β-mercaptoethanol, and 2% glusulase) and incubated overnight at 30°C, with 180 rpm shaking. The spore suspensions were then incubated at 58°C for 10 min, washed with 10 mM Tris-HCl (pH 8.0), resuspended in sterilized water, and spread on YPD plates, which were incubated for 2 to 3 days. The suspected haploid was validated using PCR (53).

### Generation of *MBP1* gene disruption cassette.

Plasmid pUG6 was used as a PCR template to generate the *loxP*–*kan*^r^–*loxP* gene disruption cassette (54). For the *MBP1* disruption experiment, two oligonucleotides (Table 2, *MBP1* gene knockout box amplification primers R and F) were used that carry a segment homologous to sequences left and right of the *loxP*–*kanMX*–*loxP* module on plasmid pUG6 at their 3′ end and a segment homologous to the *MBP1* to be disrupted at their 5′ end. The PCR conditions for the disruption cassette were as follows: 94°C for 3 min; 94°C for 30 s, 68°C decreasing (the annealing temperature of each subsequent cycle was run at 1°C less than that of the preceding cycle) for 30 s, 72°C for 2 min for 15 cycles; 94°C for 30 s, 53°C for 30 s, 72°C for 2 min for 30 cycles; and a final step of 72°C for 10 min.

**TABLE 2:** Primers used in this study

Primer use	Primer name	Primer sequence (5′–3′)	Note
*MBP1* gene knockout box amplification primers			
	R	GTGCTTAACATTCCGAGACACAACGTAAATCCCAGAAACACAAGC	Upstream of *MBP1*
		GCAGGTCGACAACCCTTAAT	Upstream of *Loxp*
	F	CAGTATATGGATACATGTAAAGTTCCTCTATTTATGTATATTTTA	Downstream of *MBP1*
		GCCACTAGTGGATCTGATATCACC	Downstream of *Loxp*
*MAT* locus verification primers			
MF**a** or MFα verification	*MAT*F	AGTCACATCAAGATCGTTTATGG	Downstream of *MAT*F
MF**a** verification	*MAT* **a**	ACTCCACTTCAAGTAAGAGTTTG	Within *MAT***a**
MFα verification	*MAT*α	GCACGGAATATGGGACTACTTCG	Within *MAT*α
*KanMX* gene integrity verification primers			
*mbp1Δ*::*Kan*^r^ verification	Ka	CAGCATCCATTAGCCGTTAG	Upstream of *MBP1*
	Kb	ATTCCGACTCGTCCAACATC	Within *KanMX*
*mbp1Δ*::*Kan*^r^ verification	Kc	AGGTCTAGAGATCTGTTTAGCTTGC	Within *KanMX*
	Kd	CACAGAAAAAGCACTGCTTACTG	Downstream of *MBP1*
*MBP1* gene integrity verification primers			
WT verification	056wa	ATGGAATACCTGCAAGATAC	Upstream of *MBP1*
	056wb	TGCAATAACACTTGTGGTAG	Within *MBP1*
WT verification	056wc	GGATCTACGAGGGGAAGCAG	Within *MBP1*
	056wd	CACTGTCTTGGGTGACGACG	Downstream of *MBP1*
Overexpression^*a*^			
pKY construction	*MBP1*-F	*GG*GGTACCGCATGTCTAACCAAATATACTCAGCG	*Kpn *I
	*MBP1*-R	*ACAT*GCATGC TCCGATAATGTTCCAAAGGAAGC	*Sph *I
pK construction	*Kan-*F	*CTA*GCTAGCTAGGTCTAGAGATCTGTTTAGCTTG	*Nhe *I
	*Kan-*R	*GGAATTC*CATATGATATTAAGGGTTCTCGAGAGCTC	*Nde *I
qRT-PCR			
*MBP1*	F	AAAAGCTGGAATACAGGCAAACGG	
	R	TGAGCCAATTCCAACCTTTCCAG	
*PDA1*	F	CTGTTGGTCAGGAGGCCATTGC	
	R	CCAGAACGGCTTTCACTGAGGC	

a The protected base is shown as italic, and the restriction enzyme cutting site is underlined.

### *MBP1* gene disruption and verification.

Mating types **a** and α of MF01 were both subjected to transformations with *loxP*–*kanMX*–*loxP*, respectively, as described previously (55). The transformants were selected by adding Geneticin (G-418 sulfate) at 300 mg/liter in solid medium or 200 mg/liter in liquid YPD. *MBP1* gene integrity verification was carried out with primers 056wa and 056wb or 056wc and 056wd, respectively (Table 2), using the following PCR protocol: 94°C for 5 min; 94°C for 30 s, 50°C for 30 s, 72°C for 2.5 min for 30 cycles; and 72°C for 10 min. Detection of the correct gene deletion of *MBP1* was performed with primers Ka and Kb or Kc and Kd, respectively (Table 2), using the following PCR protocol: 94°C for 3 min; 94°C for 30 s, 55°C decreasing (the annealing temperature of each subsequent cycle was run at 1°C less than that of the preceding cycle) for 30 s, 72°C for 2 min for 10 cycles; 94°C for 30 s, 45°C for 30 s, 72°C for 2 min for 20 cycles; and 72°C for 10 min.

### Mating.

Diploid strains were constructed as previously described (56) with modifications. Overnight strains of each haploid lacking *MBP1* were mixed and pelleted by centrifugation, and the mixtures were resuspended in appropriate YPD and subjected to incubation for 3 to 4 h without shaking. The cells were filtered onto a 0.45-μm nitrocellulose filter and then rinsed with 5 ml of YPD. The filter was placed onto a YPD plate containing 8% dextrose and incubated for 3 to 5 h. The cells were rinsed with 5 ml of YPD and subjected to centrifugation. The cells were then resuspended, plated, and incubated for 2 to 3 days. The diploid strains were selected based on their appearance and validated using PCR (53).

### Marker rescue.

The diploid *kan*^r^ yeast strain with the relevant genotype *mbp1*:: *loxP*–*kanMX*–*loxP*/*mbp1*:: *loxP*–*kanMX*–*loxP* was transformed with plasmid pSH65. The transformants were selected on YPD plates supplemented with 50 μg/ml zeocin and then incubated in YPD containing 2% galactose for more than 2 h (54). A colony lacking the marker gene was selected by plating cells on YPD and replica plating the colonies onto YPD plus G418. Cells of continuous passage culture for 10 generations for the loss of pSH65 were selected based on their ability to grow on YPD but not on YPD plus zeocin. Correct loss of the *kan*^r^ marker gene was verified by PCR using *KanMX* gene integrity verification primers (Table 2).

### Fermentation.

The method for fermentation with sucrose was as follows: 2 × 10^8^ overnight cells were inoculated into 25 ml sucrose medium (1% yeast extract, 2% Bacto peptone, and 23% sucrose, natural pH value) and grown for 32 to 34 h, followed by fermentation without shaking until 56 h, and samples were taken at 8-h intervals. The method for fermentation with molasses was as follows: 2 × 10^8^ overnight cells were grown in 10 ml molasses medium (23 degrees Brix [°Bx] molasses, 0.2% urea, 0.02% phosphoric acid [pH 3.8 to 3.9]) for 12 h, and the same molasses medium (replacing 23°BX molasses with 55°BX molasses) was supplemented (feeding), after which the cells were grown for another 24 h, followed by fermentation without shaking for 40 h. Samples were taken at 8-h intervals from feeding (0 h) to the end of fermentation. After centrifugation, the supernatants of the samples were collected and stored at −20°C until use. Frozen supernatants were thawed before the determination of ethanol or total sugar.

### Respiratory intensity assays.

Cells in the mid-log phase (8 to 10 h) were harvested by centrifugation and suspended in 1 ml YPD containing 0.1% of the respiratory indicator triphenyl tetrazolium chloride (TTC). Equal volumes of cells (1 × 10^8^/ml) were incubated with shaking (130 rpm) and statically for 4 h. Cells were harvested by centrifugation and lysed in 1 ml lysing buffer (2% Triton X-100, 1% SDS, 100 mM NaCl, 10 mM Tris, 1 mM EDTA, ∼0.3 g quartz sand [60 to 80 mesh]) for 10 min at high speed on a high-speed vortex mixer. After centrifugation, the supernatant was collected, and the precipitate was extracted with 200 μl methanol by shaking for 5 min at high speed, followed by centrifugation, after which the supernatant was combined with the previous supernatant. The presence of reduced TTC was quantified by measuring the absorbance at 490 nm (57).

### Sensitivity to cell wall inhibitors.

Detection of cell wall defects was performed as described previously (58) and was modified by replacing 0.03% sodium dodecyl sulfate (SDS) with 0.05% SDS.

### Sampling of yeast cells and preparation of crude enzyme solution and enzyme assays.

Cells were harvested by centrifugation, washed with deionized water, resuspended in solutions of Tris, EDTA, and dithiothreitol (final concentrations of 100, 5, and 2 mM, respectively [pH 7.4]) (59, 60), and then quickly frozen (−80°C) until use.

After thawing, the samples were quickly ground to a fine powder in liquid nitrogen, followed by the addition of phenylmethylsulfonyl fluoride (PMSF) at a final concentration of 1 mM and mixing immediately. After centrifugation for 5 min at 4°C (13,520 × *g*), the supernatants were collected and the protein concentrations were determined using the Bradford Protein assay kit (Beyotime Biotechnology, Beijing, China). Peroxidase (POD) activity was determined as previously described (61). Catalase (CAT) activity was determined using a catalase assay kit (visible light; Nanjing Jiancheng Bioengineering Institute, Nanjing, China) by analyzing the complex compound generated from the reaction of H_2_O_2_ and ammonium molybdate at 405 nm. Total superoxide dismutase (T-SOD) activity was analyzed using the total superoxide dismutase assay kit (hydroxylamine method; Nanjing Jiancheng Bioengineering Institute, Nanjing, China). Alcohol-dehydrogenase (ADH) activity assay was initiated by adding crude enzyme to a reaction mixture containing glycine-potassium hydroxide (pH 9), ethanol, and NAD-trihydrate (final concentrations of 50, 100, and 1 mM, respectively). After 20 min of reaction at 30°C, ADH activity was determined by measuring the increase in absorbance at 340 nm using a DU800 spectrophotometer (Beckman, USA). One enzyme unit was defined as a change of 1 in the absorbance at 340 nm per minute per milligram of protein.

### Construction of overexpression plasmid.

The plasmids (pKY) for overexpression were created by ligating the coding region of *MBP1* with *Kpn* I (5′) and *Sph* I (3′) linkers added by PCR into pYES2. The coding region of *MBP1* was amplified using the following PCR protocol: 94°C for 5 min; 94°C for 30 s, 55°C for 30 s, 72°C for 2 min 30 s for 30 cycles; and 72°C for 10 min. The control vector (pK) was created by ligating the *loxP-kanMX-loxP* disruption module with *Nhe* I (5′) and *Nde* I (3′) linkers added by PCR into pYES2. The PCR protocol was 94°C for 5 min; 94°C for 30 s, 55°C for 30 s, 72°C for 2 min for 30 cycles; and 72°C for 10 min.

### Synchronization.

Overnight cells were inoculated into starvation medium (2% dextrose, 0.05 M KH_2_PO_4_) for 1 day to induce synchronization.

### qRT-PCR analysis.

Cells were harvested by centrifugation, quickly frozen in liquid nitrogen, and stored frozen at −80°C until use or ground to a fine powder in liquid nitrogen. Total RNA was extracted using RNAiso Plus (TaKaRa Biotechnology Co., Ltd., Dalian, China). Genomic DNA digestion was carried out using Recombinant DNase I (TaKaRa) prior to purification with an RNA clean kit (Tiangen Biotech, Beijing, China). Single-stranded cDNA synthesis was performed using TransScript first-strand cDNA synthesis super mix (TransGen Biotech, Beijing, China) with approximately 3 μg RNA as a template. The products were diluted 10-fold and used as the templates for qRT-PCR amplification using SYBR Premix *Ex Taq* II (TaKaRa Biomedical Technology, Beijing, China). The qRT-PCRs were performed using an ABI 7500 system in an 8-cap strip, with each reaction mixture containing 10 μl of SYBR Premix *Ex Taq* II (2×), 0.8 μl of each forward and reverse primer (10 μM) (Table 2), 0.4 μl of ROX Reference Dye II (50×), 6 μl water, and 2 μl of diluted reverse transcription product (no more than 100 ng). The amplification protocol was 1 cycle at 95°C for 30 s and 40 cycles at 95°C for 5 s, 56°C for 30 s, and 72°C for 34 s. Fluorescence intensity was detected at 72°C in each cycle. A melting curve was generated by heating from 60°C for 1 min to 95°C for 30 s at 0.1°C/s. The specificity of the PCR product was verified by melt-curve analysis.

### Statistics.

Statistical comparisons were made using the Student’s *t* test (two-tailed, paired) using Microsoft Excel. *P* values were considered significant as follows: *, *P < *0.05; **, *P < *0.01; ***, *P < *0.001; ns (not significant), *P > *0.05.

## References

[1] Camargos CV, Moraes VD, de Oliveira LM, Guidini CZ, Ribeiro EJ, Santos LD. 2021. High gravity and very high gravity fermentation of sugarcane molasses by flocculating *Saccharomyces cerevisiae*: experimental investigation and kinetic modeling. Appl Biochem Biotechnol 193:807–821. doi:10.1007/s12010-020-03466-9. 33196971

[2] Jagtap RS, Mahajan DM, Mistry SR, Bilaiya M, Singh RK, Jain R. 2019. Improving ethanol yields in sugarcane molasses fermentation by engineering the high osmolarity glycerol pathway while maintaining osmotolerance in *Saccharomyces cerevisiae*. Appl Microbiol Biotechnol 103:1031–1042. doi:10.1007/s00253-018-9532-1. 30488283

[3] Pandey AK, Kumar M, Kumari S, Kumari P, Yusuf F, Jakeer S, Naz S, Chandna P, Bhatnagar I, Gaur NA. 2019. Evaluation of divergent yeast genera for fermentation-associated stresses and identification of a robust sugarcane distillery waste isolate *Saccharomyces cerevisiae* NGY10 for lignocellulosic ethanol production in SHF and SSF. Biotechnol Biofuels 12. doi:10.1186/s13068-019-1379-x. PMC6391804 30858877

[4] Cabañas KT, Peña-Moreno IC, Parente DC, García AB, Gutiérrez RG, de Morais MA. 2019. Selection of *Saccharomyces cerevisiae* isolates for ethanol production in the presence of inhibitors. 3 Biotech 9:6. doi:10.1007/s13205-018-1541-3. PMC6312825 30622844

[5] Cheng C, Zhang M, Xue C, Bai F, Zhao X. 2017. Development of stress tolerant *Saccharomyces cerevisiae* strains by metabolic engineering: new aspects from cell flocculation and zinc supplementation. J Biosci Bioeng 123:141–146. doi:10.1016/j.jbiosc.2016.07.021. 27576171

[6] Varela C, Pizarro F, Agosin E. 2004. Biomass content governs fermentation rate in nitrogen-deficient wine musts. Appl Environ Microbiol 70:3392–3400. doi:10.1128/AEM.70.6.3392-3400.2004. 15184136 PMC427798

[7] Coleman MC, Fish R, Block DE. 2007. Temperature-dependent kinetic model for nitrogen-limited wine fermentations. Appl Environ Microbiol 73:5875–5884. doi:10.1128/AEM.00670-07. 17616615 PMC2074923

[8] Cramer AC, Vlassides S, Block DE. 2002. Kinetic model for nitrogen-limited wine fermentations. Biotechnol Bioeng 77:49–60. doi:10.1002/bit.10133. 11745173

[9] Gasch AP, Werner-Washburne M. 2002. The genomics of yeast responses to environmental stress and starvation. Funct Integr Genomics 2:181–192. doi:10.1007/s10142-002-0058-2. 12192591

[10] Kron SJ, Styles CA, Fink GR. 1994. Symmetric cell division in pseudohyphae of the yeast *Saccharomyces cerevisiae*. Mol Biol Cell 5:1003–1022. doi:10.1091/mbc.5.9.1003. 7841518 PMC301123

[11] Gimeno CJ, Ljungdahl PO, Styles CA, Fink GR. 1992. Unipolar cell divisions in the yeast *S. cerevisiae* lead to filamentous growth: regulation by starvation and RAS. Cell 68:1077–1090. doi:10.1016/0092-8674(92)90079-R. 1547504

[12] Lambrechts MG, Bauer FF, Marmur J, Pretorius IS. 1996. Muc1, a mucin-like protein that is regulated by Mss10, is critical for pseudohyphal differentiation in yeast. Proc Natl Acad Sci USA 93:8419–8424. doi:10.1073/pnas.93.16.8419. 8710886 PMC38686

[13] Cullen PJ, Sprague GF. 2000. Glucose depletion causes haploid invasive growth in yeast. Proc Natl Acad Sci USA 97:13619–13624. doi:10.1073/pnas.240345197. 11095711 PMC17625

[14] Wu R, Chen D, Cao S, Lu Z, Huang J, Lu Q, Chen Y, Chen X, Guan N, Wei Y, Huang R. 2020. Enhanced ethanol production from sugarcane molasses by industrially engineered *Saccharomyces cerevisiae* via replacement of the *PHO4* gene. RSC Adv 10:2267–2276. doi:10.1039/C9RA08673K. 35494577 PMC9048610

[15] Jetti KD, Gns RR, Garlapati D, Nammi SK. 2019. Improved ethanol productivity and ethanol tolerance through genome shuffling of *Saccharomyces cerevisiae* and *Pichia stipitis*. Int Microbiol 22:247–254. doi:10.1007/s10123-018-00044-2. 30810988

[16] Jeong D, Ye S, Park H, Kim SR. 2020. Simultaneous fermentation of galacturonic acid and five-carbon sugars by engineered *Saccharomyces cerevisiae*. Bioresour Technol 295:122259. doi:10.1016/j.biortech.2019.122259. 31639627

[17] Hoang Nguyen Tran P, Ko JK, Gong G, Um Y, Lee SM. 2020. Improved simultaneous co-fermentation of glucose and xylose by *Saccharomyces cerevisiae* for efficient lignocellulosic biorefinery. Biotechnol Biofuels 13:1019–1641. doi:10.1186/s13068-019-1641-2. PMC6975041 31993090

[18] Ruchala J, Kurylenko OO, Dmytruk KV, Sibirny AA. 2020. Construction of advanced producers of first- and second-generation ethanol in *Saccharomyces cerevisiae* and selected species of non-conventional yeasts (*Scheffersomyces stipitis*, *Ogataea polymorpha*). J Ind Microbiol Biotechnol 47:109–132. doi:10.1007/s10295-019-02242-x. 31637550 PMC6970964

[19] Peng B, Shen Y, Li X, Chen X, Hou J, Bao X. 2012. Improvement of xylose fermentation in respiratory-deficient xylose-fermenting *Saccharomyces cerevisiae*. Metab Eng 14:9–18. doi:10.1016/j.ymben.2011.12.001. 22178745

[20] Zheng L, Wei S, Wu M, Zhu X, Bao X, Hou J, Liu W, Shen Y. 2020. Improving xylose fermentation in *Saccharomyces cerevisiae* by expressing nuclear-localized hexokinase 2. Microorganisms 8:856. doi:10.3390/microorganisms8060856. 32517148 PMC7356972

[21] Liu H, Styles CA, Fink GR. 1996. *Saccharomyces cerevisiae* S288C has a mutation in *FLO8*, a gene required for filamentous growth. Genetics 144:967–978. doi:10.1093/genetics/144.3.967. 8913742 PMC1207636

[22] Liu ZL, Wang X, Weber SA. 2018. Tolerant industrial yeast *Saccharomyces cerevisiae* possess a more robust cell wall integrity signaling pathway against 2-furaldehyde and 5-(hydroxymethyl)-2-furaldehyde. J Biotechnol 276–277:15–24. doi:10.1016/j.jbiotec.2018.04.002. 29665400

[23] Nijland JG, Shin HY, Boender LGM, de Waal PP, Klaassen P, Driessen AJM. 2017. Improved xylose metabolism by a CYC8 mutant of *Saccharomyces cerevisiae*. Appl Environ Microbiol 83:e00095-17. doi:10.1128/AEM.00095-17. 28363963 PMC5440722

[24] Bean JM, Siggia ED, Cross FR. 2005. High functional overlap between MluI cell-cycle box binding factor and Swi4/6 cell-cycle box binding factor in the G1/S transcriptional program in *Saccharomyces cerevisiae*. Genetics 171:49–61. doi:10.1534/genetics.105.044560. 15965243 PMC1456534

[25] Porter SE, Washburn TM, Chang M, Jaehning JA. 2002. The yeast pafl-rNA polymerase II complex is required for full expression of a subset of cell cycle-regulated genes. Eukaryot Cell 1:830–842. doi:10.1128/EC.1.5.830-842.2002. 12455700 PMC126743

[26] Neurohr GE, Terry RL, Lengefeld J, Bonney M, Brittingham GP, Moretto F, Miettinen TP, Vaites LP, Soares LM, Paulo JA, Harper JW, Buratowski S, Manalis S, van Werven FJ, Holt LJ, Amon A. 2019. Excessive cell growth causes cytoplasm dilution and contributes to senescence. Cell 176:1083–1097.e18. doi:10.1016/j.cell.2019.01.018. 30739799 PMC6386581

[27] Jorgensen P, Nishikawa JL, Breitkreutz B-J, Tyers M. 2002. Systematic identification of pathways that couple cell growth and division in yeast. Science 297:395–400. doi:10.1126/science.1070850. 12089449

[28] Koch C, Moll T, Neuberg M, Ahorn H, Nasmyth K. 1993. A role for the transcription factors Mbp1 and Swi4 in progression from G1 to S phase. Science 261:1551–1557. doi:10.1126/science.8372350. 8372350

[29] Shaner L, Gibney PA, Morano KA. 2008. The Hsp110 protein chaperone Sse1 is required for yeast cell wall integrity and morphogenesis. Curr Genet 54:1–11. doi:10.1007/s00294-008-0193-y. 18478233 PMC5635425

[30] Hussein B, Huang H, Glory A, Osmani A, Kaminskyj S, Nantel A, Bachewich C. 2011. G1/S transcription factor orthologues Swi4p and Swi6p are important but not essential for cell proliferation and influence hyphal development in the fungal pathogen *Candida albicans*. Eukaryot Cell 10:384–397. doi:10.1128/EC.00278-10. 21257795 PMC3067467

[31] Bouquin N, Johnson AL, Morgan BA, Johnston LH. 1999. Association of the cell cycle transcription factor mbp1 with the skn7 response regulator in budding yeast. Mol Biol Cell 10:3389–3400. doi:10.1091/mbc.10.10.3389. 10512874 PMC25606

[32] Cullen PJ, Sprague GF. 2012. The regulation of filamentous growth in yeast. Genetics 190:23–49. doi:10.1534/genetics.111.127456. 22219507 PMC3249369

[33] Schwob E, Nasmyth K. 1993. CLB5 and CLB6, a new pair of B cyclins involved in DNA replication in *Saccharomyces cerevisiae*. Genes Dev 7:1160–1175. doi:10.1101/gad.7.7a.1160. 8319908

[34] Vandermeulen MD, Cullen PJ. 2020. New aspects of invasive growth regulation identified by functional profiling of MAPK pathway targets in *Saccharomyces cerevisiae*. Genetics 216:95–116. doi:10.1534/genetics.120.303369. 32665277 PMC7463291

[35] Loeb JD, Kerentseva TA, Pan T, Sepulveda-Becerra M, Liu H. 1999. *Saccharomyces cerevisiae* G1 cyclins are differentially involved in invasive and pseudohyphal growth independent of the filamentation mitogen-activated protein kinase pathway. Genetics 153:1535–1546. doi:10.1093/genetics/153.4.1535. 10581264 PMC1460854

[36] Colomina N, Ferrezuelo F, Verges E, Aldea M, Gari E. 2009. Whi3 regulates morphogenesis in budding yeast by enhancing Cdk functions in apical growth. Cell Cycle 8:1912–1920. doi:10.4161/cc.8.12.8740. 19440046

[37] Dorsey S, Tollis S, Cheng J, Black L, Notley S, Tyers M, Royer CA. 2018. G1/S transcription factor copy number is a growth-dependent determinant of cell cycle commitment in Yeast. Cell Syst 6:539–554. doi:10.1016/j.cels.2018.04.012. 29792825

[38] Horak CE, Luscombe NM, Qian J, Bertone P, Piccirrillo S, Gerstein M, Snyder M. 2002. Complex transcriptional circuitry at the G1/S transition in *Saccharomyces cerevisiae*. Genes Dev 16:3017–3033. doi:10.1101/gad.1039602. 12464632 PMC187492

[39] Wijnen H, Landman A, Futcher B. 2002. The G(1) cyclin Cln3 promotes cell cycle entry via the transcription factor Swi6. Mol Cell Biol 22:4402–4418. doi:10.1128/MCB.22.12.4402-4418.2002. 12024050 PMC133883

[40] Hendler A, Medina EM, Kishkevich A, Abu-Qarn M, Klier S, Buchler NE, de Bruin RAM, Aharoni A. 2017. Gene duplication and co-evolution of G1/S transcription factor specificity in fungi are essential for optimizing cell fitness. PLoS Genet 13:e1006778. doi:10.1371/journal.pgen.1006778. 28505153 PMC5448814

[41] Roberts RL, Fink GR. 1994. Elements of a single MAP kinase cascade in *Saccharomyces cerevisiae* mediate two developmental programs in the same cell type: mating and invasive growth. Genes Dev 8:2974–2985. doi:10.1101/gad.8.24.2974. 8001818

[42] Saito H. 2010. Regulation of cross-talk in yeast MAPK signaling pathways. Curr Opin Microbiol 13:677–683. doi:10.1016/j.mib.2010.09.001. 20880736

[43] Olivares-Marin IK, Gonzhat PJ. 2017. *Saccharomyces cerevisiae* exponential growth kinetics in batch culture to analyze respiratory and fermentative metabolism. J VisExp 139:e58192. doi:10.3791/58192. PMC6235381 30320748

[44] Kumar A, Dandekar JU, Bhat PJ. 2017. Fermentative metabolism impedes p53-dependent apoptosis in a Crabtree-positive but not in Crabtree-negative yeast. J Biosci 42:585–601. doi:10.1007/s12038-017-9717-2. 29229877

[45] Martinez‐Ortiz C, Carrillo‐Garmendia A, Correa‐Romero BF, Canizal‐García M, González‐Hernández JC, Regalado‐Gonzalez C, Olivares‐Marin IK, Madrigal‐Perez LA. 2019. SNF1 controls the glycolytic flux and mitochondrial respiration. Yeast 36:487–494. doi:10.1002/yea.3399. 31074533

[46] Yi C, Tong J, Lu P, Wang Y, Zhang J, Sun C, Yuan K, Xue R, Zou B, Li N, Xiao S, Dai C, Huang Y, Xu L, Li L, Chen S, Miao D, Deng H, Li H, Yu L. 2017. Formation of a Snf1-Mec1-Atg1 module on mitochondria governs energy deprivation-induced autophagy by regulating mitochondrial respiration. Dev Cell 41:59–71. doi:10.1016/j.devcel.2017.03.007. 28399401

[47] Busnelli S, Tripodi F, Nicastro R, Cirulli C, Tedeschi G, Pagliarin R, Alberghina L, Coccetti P. 2013. Snf1/AMPK promotes SBF and MBF-dependent transcription in budding yeast. Biochim Biophys Acta 1833:3254–3264. doi:10.1016/j.bbamcr.2013.09.014. 24084603

[48] Busti S, Coccetti P, Alberghina L, Vanoni M. 2010. Glucose signaling-mediated coordination of cell growth and cell cycle in *Saccharomyces cerevisiae*. Sensors (Basel) 10:6195–6240. doi:10.3390/s100606195. 22219709 PMC3247754

[49] Pessina S, Tsiarentsyeva V, Busnelli S, Vanoni M, Alberghina L, Coccetti P. 2010. Snf1/AMPK promotes S-phase entrance by controlling CLB5 transcription in budding yeast. Cell Cycle 9:2189–2200. doi:10.4161/cc.9.11.11847. 20505334

[50] Backhaus K, Rippert D, Heilmann CJ, Sorgo AG, de Koster CG, Klis FM, Rodicio R, Heinisch JJ. 2013. Mutations in SNF1 complex genes affect yeast cell wall strength. Eur J Cell Biol 92:383–395. doi:10.1016/j.ejcb.2014.01.001. 24486034

[51] Pham TH, Clemente JC, Satou K, Ho TB. 2005. Computational discovery of transcriptional regulatory rules. Bioinformatics 21:ii101–ii107. doi:10.1093/bioinformatics/bti1117. 16204087

[52] Koprowski P, Fikus MU, Dzierzbicki P, Mieczkowski P, Lazowska J, Ciesla Z. 2003. Enhanced expression of the DNA damage-inducible gene DIN7 results in increased mutagenesis of mitochondrial DNA in *Saccharomyces cerevisiae*. Mol Genet Genomics 269:632–639. doi:10.1007/s00438-003-0873-8. 12827502

[53] Huxley C, Green ED, Dunham I. 1990. Rapid assessment of *S. cerevisiae* mating type by PCR. Trends Genet 6:9168–9525. 10.1016/0168-9525(90)90190-h 2238077

[54] Güldener U, Heck S, Fielder T, Beinhauer J, Hegemann JH. 1996. A new efficient gene disruption cassette for repeated use in budding yeast. Nucleic Acids Res 24:2519–2524. doi:10.1093/nar/24.13.2519. PMC145975 8692690

[55] Gietz RD, Schiestl RH. 2007. High-efficiency yeast transformation using the LiAc/SS carrier DNA/PEG method. Nat Protoc 2:31–34. doi:10.1038/nprot.2007.13. 17401334

[56] Shuster JR. 1982. Mating-defective ste mutations are suppressed by cell division cycle start mutations in *Saccharomyces cerevisiae*. Mol Cell Biol 2:1052–1063. doi:10.1128/mcb.2.9.1052-1063.1982. 6757719 PMC369898

[57] Burmølle M, Webb JS, Rao D, Hansen LH, Sørensen SJ, Kjelleberg S. 2006. Enhanced biofilm formation and increased resistance to antimicrobial agents and bacterial invasion are caused by synergistic interactions in multispecies biofilms. Appl Environ Microbiol 72:3916–3923. doi:10.1128/AEM.03022-05. 16751497 PMC1489630

[58] Baker LG, Specht CA, Donlin MJ, Lodge JK. 2007. Chitosan, the deacetylated form of chitin, is necessary for cell wall integrity in *Cryptococcus neoformans*. Eukaryot Cell 6:855–867. doi:10.1128/EC.00399-06. 17400891 PMC1899242

[59] Viegas CA, Sa-Correia I. 1991. Activation of plasma membrane ATPase of *Saccharomyces cerevisiae* by octanoic acid. J Gen Microbiol 137:645–651. doi:10.1099/00221287-137-3-645. 1827836

[60] Rosa MF, Sá-Correia I. 1991. *In vivo* activation by ethanol of plasma membrane ATPase of *Saccharomyces cerevisiae*. Appl Environ Microbiol 57:830–835. doi:10.1128/aem.57.3.830-835.1991. 1645512 PMC182802

[61] Heinzkill M, Bech L, Halkier T, Schneider P, Anke T. 1998. Characterization of laccases and peroxidases from wood-rotting fungi (family Coprinaceae). Appl Environ Microbiol 64:1601–1606. doi:10.1128/AEM.64.5.1601-1606.1998. 9572923 PMC106202

[62] Miller GL. 1959. Use of dinitrosalicylic acid reagent for determination of reducing sugar. Analytical Biochemistry 31:426–428.

[63] Livak KJ, Schmittgen TD. 2001. Analysis of relative gene expression data using real-time quantitative PCR and the 2^-ΔΔCT^ method. Methods 25:402–408. doi:10.1006/meth.2001.1262. 11846609

[64] Shima J, Hino A, Yamada-Iyo C, Suzuki Y, Nakajima R, Watanabe H, Mori K, Takano H. 1999. Stress tolerance in doughs of *Saccharomyces cerevisiae* trehalase mutants derived from commercial baker’s yeast. Appl Environ Microbiol 65:2841–2846. doi:10.1128/AEM.65.7.2841-2846.1999. 10388673 PMC91426

[65] Da Costa BLV, Basso TO, Raghavendran V, Gombert AK. 2018. Anaerobiosis revisited: growth of *Saccharomyces cerevisiae* under extremely low oxygen availability. Appl Microbiol Biotechnol 102:2101–2116. doi:10.1007/s00253-017-8732-4. 29397429

